# The Effects of Different Media on Shoot Proliferation From the Shoot Tip of *Aloe vera L.*

**Published:** 2013-05-04

**Authors:** Mohammad Hosein Daneshvar, Noorolah Moallemi, Nazanin Abdolah Zadeh

**Affiliations:** 1 Department of Horticulture, Faculty of Agriculture, Ramin Agricultural and Natural Resources University, Ahvaz, IR Iran; 2 Department of Horticulture, Faculty of Agriculture, Shahid Chamran University of Ahvaz , Ahvaz, IR Iran

**Keywords:** Plant Shoots, Benzylaminopurine, Plants, Medicinal

## Abstract

**Background:**

*Aloe vera L.* is an important pharmaceutical plant from which several medicinal and cosmetic compounds are extracted. Aloe is naturally propagated through offset, which is a slow and expensive labor cost method with low economical income.

**Objectives:**

In this study, the effect of different media on shoot proliferation of the shoot tip of *Aloe vera L.* was investigated.

**Materials and Methods:**

In vitro techniques are some of the suggested methods for rapid propagation of Aloe. In this experiment, the shoot tips of mother plants were grown in a greenhouse. After surface sterilization of the explants, they were cultured on Murashige and Skoog (1962) (MS) medium containing different concentrations of kinetin and naphthaleneacetic acid (NAA). The experiment was carried out in the form of a randomized complete design with three replications.

**Results:**

The results showed that MS media containing 1.5 mg/L kinetin along with 0.15 or 0.3 mg/L NAA produced the highest percentage of proliferated shoots. In addition, the percentage of proliferated shoots in MS medium containing 2.0 or 2.5 mg/L benzylaminopurine (BAP) + 0.15 mg/L NAA was significantly higher than the other treatments.

**Conclusions:**

Analysis of the interactive effects of NAA, kinetin and BAP on shoot proliferation showed that most of the proliferated shoots produced in MS medium containing 1.0 mg/L BAP + 1.0 mg/L kinetin + 0.15 mg/L NAA were significantly different from other treatments. Rooting quality was greater in MS media containing 1.0 mg/L IBA than a 1.0 mg/L NAA treatment.

## 1. Background

*Aloe vera L.* is an important pharmaceutical plant belonging to the Liliaceae family. Several medicinal and cosmetic compounds are extracted from this plant. Its positive effects include healing wounds and burns, reducing the blood glucose levels, preventing UV ray damage, antibacterial and antifungal effects. This plant is naturally propagated through axillary shoots (offsets) using a slow method that has an expensive labor cost with low economical income (1, 2). The male - sterility of this plant makes its propagation via seed extremely difficult (1). Therefore, in vitro techniques are used for rapid propagation of Aloe (2-4). The effect of the type and concentration of plant growth regulators (1, 5-8), various media (9-11) and the type of explant (3, 12, 13) on in vitro propagation of Aloe have been studied.

## 2. Objectives

In this study, the effect of different media on shoot proliferation of the shoot tip of *Aloe vera L.* was investigated.

## 3. Materials and Methods

In this study, the plant of Aloe barbadensis syn. *Aloe vera L.* was used as the source of the explant.

### 3.1. Preparation of Explants

The shoot tips of greenhouse-grown mother plants were used for the experiment. The explants were transferred to a tissue culture laboratory. They were immersed in a liter of tap water containing 30 drops of washing liquid for 5 minutes and then washed with tap water for 15 minutes. The meristem and two young leaves around the shoot tip were separated and washed using the same method. Following the tap water rinse, the explants were kept in sterile distilled water until undergoing surface sterilization. In order to do surface sterilization, the shoot tips were immersed in 70% ethanol for 60 seconds, soaked in 20% sodium hypochlorite (NaOCl) for 15 minutes and then followed by three 5-minute rinses in distilled water. After separating the meristem and the two young leaves around it, the explants (meristem and two leaves) were immersed in 70% ethanol for 3 seconds. They were then soaked in NaOCl (2.0%) for 45 seconds followed by three 5-minute rinses in sterile distilled water. The explants were kept in sterile distilled water until starting the experiment (maximum of 15 minutes). The shoot tip explants were cultured in modified Murashige and Skoog (1962) medium containing kinetin (0, 0.5, 1.0, 1.5 and 2.0 mg/L), BAP (0, 0.5, 1.0, 1.5, 2.0, 2.5, 3.0, 5.0 and 10.0 mg/L) with or without a combination of auxins, such as NAA (0, 0.075, 0.15, 0.3 and 0.6 mg/L), 2,4-D (0, 0.06, 0.125 and 0.25 mg/L) and IBA (0.1 and 0.2 mg/L). Cultures were incubated at 25° C ± 2 with a 16/8 hour (day/night) photoperiod and an irradiance of 1500 LUX using Sylvania cool white fluorescent tubes. For cultures incubated in the dark, light was excluded by wrapping the trays of jars with black polyethylene bags. After 12 weeks, the number of proliferated shoots and the percentage of proliferated media were measured.

### 3.2. Statistical Analysis

Each experiment was carried out in the form of a randomized complete design with three replications (each replicate contained 10 jar samples). The mean number of shoots was compared in 5% level with Duncan’s multiple range tests.

### 3.3. Rooting

Proliferated shoots were transferred into MS media containing different concentrations of IBA (0, 0.1, 0.5 and 1.0 mg/L) and NAA (0.5 and 1.0 mg/L). In addition, the mean number and the length of the roots were measured.

## 4. Results

In media without cytokinin, the explant produced either a callus or single shoot; given that the roots were produced, the result of this experiment was not considered using statistical analysis.

### 4.1. The First Experiment

In MS medium containing 10 mg/L BAP and 0.1 mg/L IBA, the explants turned brownish and died after 1-2 weeks. After a reduction in BAP concentration to 5.0 mg/L and an increase in the IBA concentration to 0.2 mg/L, explants again turned brown and died.

### 4.2. The Second Experiment

The results of the interactive effects of kinetin and 2,4-D showed that in MS medium containing kinetin (1.0 mg/L) and 2,4-D (0.06 mg/L), the number of shoots (3.68) and the mean number of cultures with proliferated shoots (76.67%) were significantly more than other treatments (Table 1).

**Table 1.** Effect of Combination of Kinetin and 2,4-D a on Shoot Tip Proliferation of *Aloe vera L.* b

Plant Growth Regulator, mg/L			
Kinetin	2-4-D	Mean Shoot, No.	Media for Proliferation, %
**0.5**	0.25	0.0 e	0.0 d
**1.0**	0.125	0.0 e	0.0 d
**0.5**	0.06	2.36 c	60 b
**1.0**	0.06	3.68 a	76.67 a
** 1.5**	0.06	1.52 d	63.33 b
**0.5**	0.0	0.0 e	0.0 d
**1.0**	0.0	1.92 d	43.33 c
**1.5**	0.0	3.19 b	53.32 b, c

^a^Abbreviations: 1-2, 4-dichlorophenoxyacetic acid

^b^Values Followed by the Same Letter in Each Column Are Not Significantly Different (P < 0.05) Using DMRT

### 4.3. The Third Experiment

Analysis of the effect of different concentrations of kinetin and NAA showed that the number of proliferated shoots in MS medium containing 1.5 mg/L kinetin + 0.15 mg/L NAA had no statistically significant difference from the number of proliferated shoots in MS media containing kinetin (1.5 mg/L) + NAA (0.3 mg/L), and/or MS media containing 0.15 mg/L NAA along with kinetin 1.5 or 2.0 mg/L, but it was more than others. The percentage of proliferated shoots produced in MS medium containing 1.5 mg/L kinetin along with 0.15 or 0.3 mg/L NAA had a statistically significant difference from other treatments (Table 2).

**Table 2.** Effect of Combination of Kinetin and NAA a on Shoot Proliferation of *Aloe vera L.* b

Plant Growth Regulator, mg/L			
Kinetin	NAA	Mean Shoot, No.	Media for Proliferation, %
**0.5**	0.3	3.71 c, d, f	66.67 d
**1.0**	0.3	4.29 b, c, d	76.67 b, c
**1.5**	0.3	6.15 a, b	96.67 a
**2.0**	0.3	5.0 b, c, d	80.0 b, c
**0.5**	0.15	4.5 b, c, d	73.33 c, d
**1.0**	0.15	5.39 a, b, c	80.0 b, c
**1.5**	0.15	7.11 a	100 a
**2.0**	0.15	6.05 a, b	83.33 b
**0.5**	0.0	0.0 f	0.0 f
**1.0**	0.0	2.24 e	43.33 f
**1.5**	0.0	3.19 d, e	53.33 e
**2.0**	0.0	2.36 e	56.67 e

^a^Abbreviations: NAA, 1-naphthaleneacetic acid

^b^Values Followed by the Same Letter in Each Column Are Not Significantly Different (P < 0.05) Using DMRT

### 4.4. The Fourth Experiment

Analysis of the effects of different concentrations of BAP + 0.15 mg/L NAA in MS medium showed that the highest number of proliferated shoots (28.47) was obtained in treatment with concentrations of 2.5 mg/L BAP + 0.15 mg/L NAA, which had a statistically significant difference from other treatments. The percentage of proliferated shoots with concentrations of 2.0 or 2.5 mg/L BAP + 0.15 mg/L NAA was significantly more than other treatments (Table 3).

**Table 3.** Effect of Combination of BAP and NAA on shoot proliferation of *Aloe vera L.* a^, ^b

Plant Growth Regulator, mg/L			
BAP	NAA	Mean Shoot, No.	Media for Proliferation, %
**0.5**	0.15	9.21 e	80.0 c
**1.0**	0.15	10.32 e	83.33 c
**1.5**	0.15	14.92 d	86.67 b, c
**2.0**	0.15	20.43 b	96.67 a, b
**2.5**	0.15	28.47 a	100 a
**3.0**	0.15	17.0 c	90.0 a, b, c

^a^Abbreviations: BAP, benzylaminopurine; NAA, 1-naphthaleneacetic acid

^b^Values Followed by the Same Letter in Each Column Are Not Significantly Different (P < 0.05) Using DMRT

### 4.5. The Fifth Experiment

**Figure 1.**
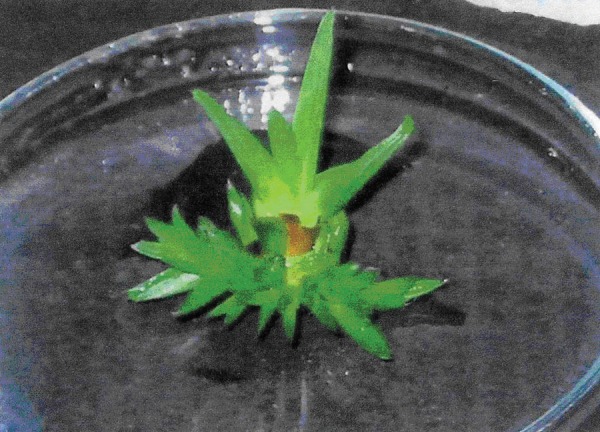
The Proliferated Shoots in MS Medium Containing 2.0 mg/L BAP + 0.15 mg/L NAA

Analysis of the interactive effect of NAA, kinetin and BAP on shoot proliferation showed that most of the proliferated shoots produced in MS medium containing 1.0 mg/L BAP + 1.0 mg/L kinetin + 0.15 mg/L NAA had a statistically significant difference from other treatments (Table 4). The percentage of proliferation in media in all of the treatments (80-97%) had no significant differences from each other (Figure 1).

**Table 4.** Effect of Combination of BAP + kinetin + NAA on Shoot Proliferation of *Aloe vera L.* a^, ^b

Plant Growth Regulator, mg/L				
BAP	Kinetin	NAA	Mean Shoot, No.	Media for Proliferation, %
**0.5**	0.5	0.15	6.6 e	80.0 a
**1.0**	1.0	0.15	12.53 a	96.67 a
**1.5**	1.5	0.15	7.12 b	86.67 a
**1.0**	1.0	0.075	7.34 c	90.0 a
**1.0**	1.0	0.3	9.17 c	86.67 a
**1.0**	1.0	0.6	8.19 d	83.33 a

^a^Abbreviations: BAP, benzylaminopurine; NAA, 1-naphthaleneacetic acid

^b^Values Followed by the Same Letter in Each Column Are Not Significantly Different (P < 0.05) Using DMRT

**Figure 2.**
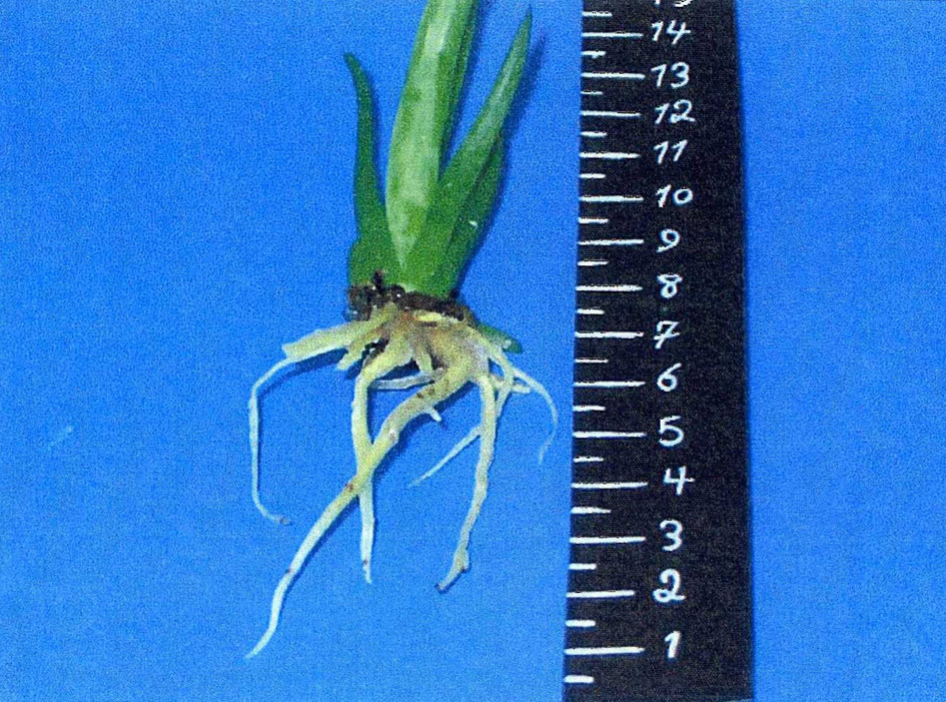
The Rooting of Proliferated Shoots in MS Medium Containing 0.5 mg/L IBA

**Figure 3.**
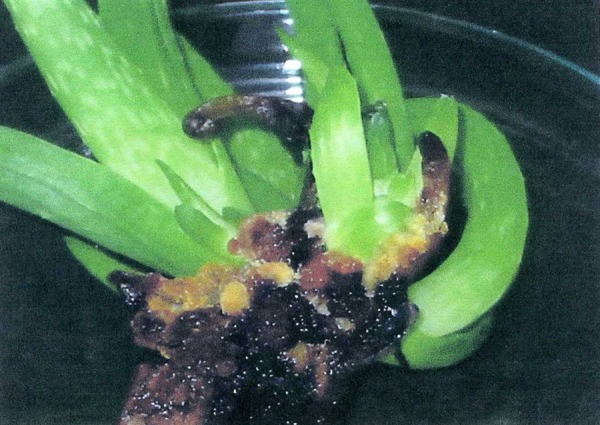
The Rooting of Proliferated Shoots in MS Medium Containing 0.5 mg/L NAA With a Callus Formed

Comparing the effect of MS media containing IBA and NAA on rooting of proliferated shoots revealed that the rooting mean (6.8) in a concentration of 0.5 mg/L NAA was significantly more than other treatments (Table 5 and Figure 2). The greatest root length of proliferated shoots was observed with treatment of 1.0 mg/L IBA that statistically did not have any noticeable difference compared to the auxin-free media. Rooting quality in IBA treatment was greater than the NAA treatment, because the explants in IBA treatment produced long, fine and ramified roots, whereas the joint of the roots and the ends of the callus shoots with NAA treatment were not ramified (Figure 3).

**Table 5.** Effect of Media on Root Number and Mean Root Length / Plantlet of *Aloe vera L.* a

Rooting Media (MS b + Auxin), g	Mean Root, No. / Plantlet	Mean Root Length / Plantlet
**IIBA 0.1, mg/L**	5.18 c, d	5.18 b
**IIBA 0.5, mg/L**	5.4 c	4.15 c
**IIBA 1.0, mg/L**	2.67 e	6.42 a
**MS, Free Auxin**	4.9 d	5.71 a, b
**NAA 0.5, mg/L**	6.8 a	5.56 b
**NAA 1.0, mg/L**	6.13 b	4.19 c

^b^Abbreviations: MS, murashige and skoog

^a^Values Followed by the Same Letter in Each Column Are Not Significantly Different (P < 0.05) Using DMRT

## 5. Discussion

In this study, the effect of cytokinins on shoot proliferation of *Aloe vera L.* has been surveyed. In media free of cytokinin, the explants produced mostly callus and / or a single shoot along with rhizogenesis. Therefore, they have not been considered for statistical analysis. In various studies, the importance of plant growth regulators on axillary bud or shoot tip propagation has been emphasized (14-18). The necessity of using both cytokinin and auxin for shoot proliferation has been reported by others (3, 19, 20). It has also been shown that the suitable ratio of cytokinin to auxin for proliferation of shoot tips of *Aloe vera L.* was 10:1. The same result has been reported by a number of other researchers (8, 14, 21). In numerous studies, the positive effect of BAP alone (16) or BAP + IAA (18) on shoot proliferation of *Aloe vera L.* has been surveyed. The results of this experiment regarding the positive effect of BAP + kinetin+ NAA (Table 4) or BAP + NAA (Table 3) on shoot proliferation of *Aloe vera L.* reinforces the conclusion of Budhiani (2001) which showed that BAP + NAA has a positive effect on Aloe shoot proliferation. However, the concentrations of both plant growth regulators differ from the concentrations reported by Budhiani (2001). Similarly, it has been suggested that in comparison to other cytokinins, BAP has a greater effect on Aloe shoot proliferation (21-23). In this experiment, the shoot tip explant was used for shoot proliferation despite the fact that Gui et al. (1990) has suggested that the greatest number of proliferated shoots in *Aloe vera L.* was achieved from stem explants without the shoot tip (24). The results of this experiment coincide with the report of Hoseini and Parsa (2007) which showed the apical dominance in various media have the best potential for regeneration (25). In this experiment, the shoot tip explants in MS media containing different concentrations of BAP + kinetin + NAA had greater effect than BAP+ NAA on shoot proliferation (Tables 3 and 4). Hosseini and Parsa (2007) have also emphasized the positive effect of the simultaneous use of kinetin and BAP on Aloe shoot proliferation (25). The results of this experiment regarding the effect of IBA and NAA on shoot rhizogenesis showed that application of NAA in an in vitro condition has a better effect on root formation. Although other studies have shown that NAA and \ or IBA are the most common auxins used for root formation in vitro (26), the results of the present study are similar to the report by Ahmad et al. (2007) that in MS medium, only NAA is the best auxin for rhizogenesis of Aloe explants (27). This is while Hashem Abadi and Kaviani (2008) have reported that Aloe explants had the best rhizogenesis in media containing NAA and BAP (21). In addition, the present study has also shown that the quality of produced roots in media containing IBA is much better. Further studies are needed to investigate the percentage of rhizogenesis and the root length of the produced roots. Furthermore, the comparison of the effect of various kinds of auxins on various indices of rhizogenesis must also be investigated.
